# Highly sensitive and robust peroxidase-like activity of Au–Pt core/shell nanorod-antigen conjugates for measles virus diagnosis

**DOI:** 10.1186/s12951-018-0371-0

**Published:** 2018-05-02

**Authors:** Lin Long, Jianbo Liu, Kaishun Lu, Tao Zhang, Yunqing Xie, Yinglu Ji, Xiaochun Wu

**Affiliations:** 1 Zaozhuang Municipal Center for Disease Control and Prevention, Zaozhuang, 277100 China; 2 College of Opto-electronic Engineering, Zaozhuang University, Zaozhuang, 277160 China; 3 CAS Key Laboratory of Standardization and Measurement for Nanotechnology, National Center for Nanoscience and Technology, Beijing, 100190 China

**Keywords:** Gold nanorods, Platinum, Conjugate, Peroxidase mimic, Enzyme-linked immunosorbent assay (ELISA), Virus diagnosis

## Abstract

**Background:**

As a promising candidate for artificial enzymes, catalytically active nanomaterials show several advantages over natural enzymes, such as controlled synthesis at low cost, tunability of catalytic activities, and high stability under stringent conditions. Rod-shaped Au–Pt core/shell nanoparticles (Au@Pt NRs), prepared by Au nanorod-mediated growth, exhibit peroxidase-like activities and could serve as an inexpensive replacement for horseradish peroxidase, with potential applications in various bio-detections. The determination of measles virus is accomplished by a capture-enzyme-linked immunosorbent assay (ELISA) using Au@Pt NR-antigen conjugates.

**Results:**

Based on the enhanced catalytic properties of this nanozyme probe, a linear response was observed up to 10 ng/mL measles IgM antibodies in human serum, which is 1000 times more sensitive than commercial ELISA.

**Conclusions:**

Hence, these findings provide positive proof of concept for the potential of Au@Pt NR-antigen conjugates in the development of colorimetric biosensors that are simple, robust, and cost-effective.

**Electronic supplementary material:**

The online version of this article (10.1186/s12951-018-0371-0) contains supplementary material, which is available to authorized users.

## Background

As a natural product, enzymes are extremely efficient at catalyzing a variety of reactions with high substrate specificity under mild reaction conditions [1]. For example, enzyme-linked immunosorbent assay (ELISA) is the most widely accepted and powerful method for virus detection. This method commonly uses a horseradish peroxidase (HRP)-labeled immunoreagents to realize the amplification of detection signals and the identification of target molecules [2]. In spite of the high catalytic efficiency, natural enzymes have critical limitations for industrial application, such as low stability in harsh conditions (temperature and pH) and relatively high costs for preparation, purification, and storage. Additionally, enzymatic labeling always involves time-consuming preparation and sophisticated purification processes [3]. Therefore, over the past few decades, researchers have made an intense effort to develop artificial enzymes for a wide range of applications [4].

The rapid development of nanotechnology over the past decade has allowed us to witness a new perspective of conventional heterogeneous catalysts, thus offering great opportunities for developing nanomaterial-based artificial enzymes (nanozymes) [5]. To date, many nanomaterials, such as magnetic nanoparticles (NPs) [6], transition metal chalcogenide nanostructures [7], graphene oxide [8], and noble metal nanostructures [9], have been discovered to possess unique enzyme-mimic catalytic activities and show promising potentials in various biological assays. Compared with natural enzymes, nanozymes are advantageous in several aspects, such as low cost, ease of mass production, robustness in harsh environments, high stability, long-term storage and large surface area for further modification and bioconjugation [10]. In addition, as the properties of nanoscale materials are often dependent on size, structure, dopant, morphology and surface modification, the catalytic activity of nanozymes is readily tunable by controlling these parameters [11]. For instance, combination of biomolecules with NPs provides interesting tools for improvements in traditional ELISA [12, 13].

**Scheme 1:**
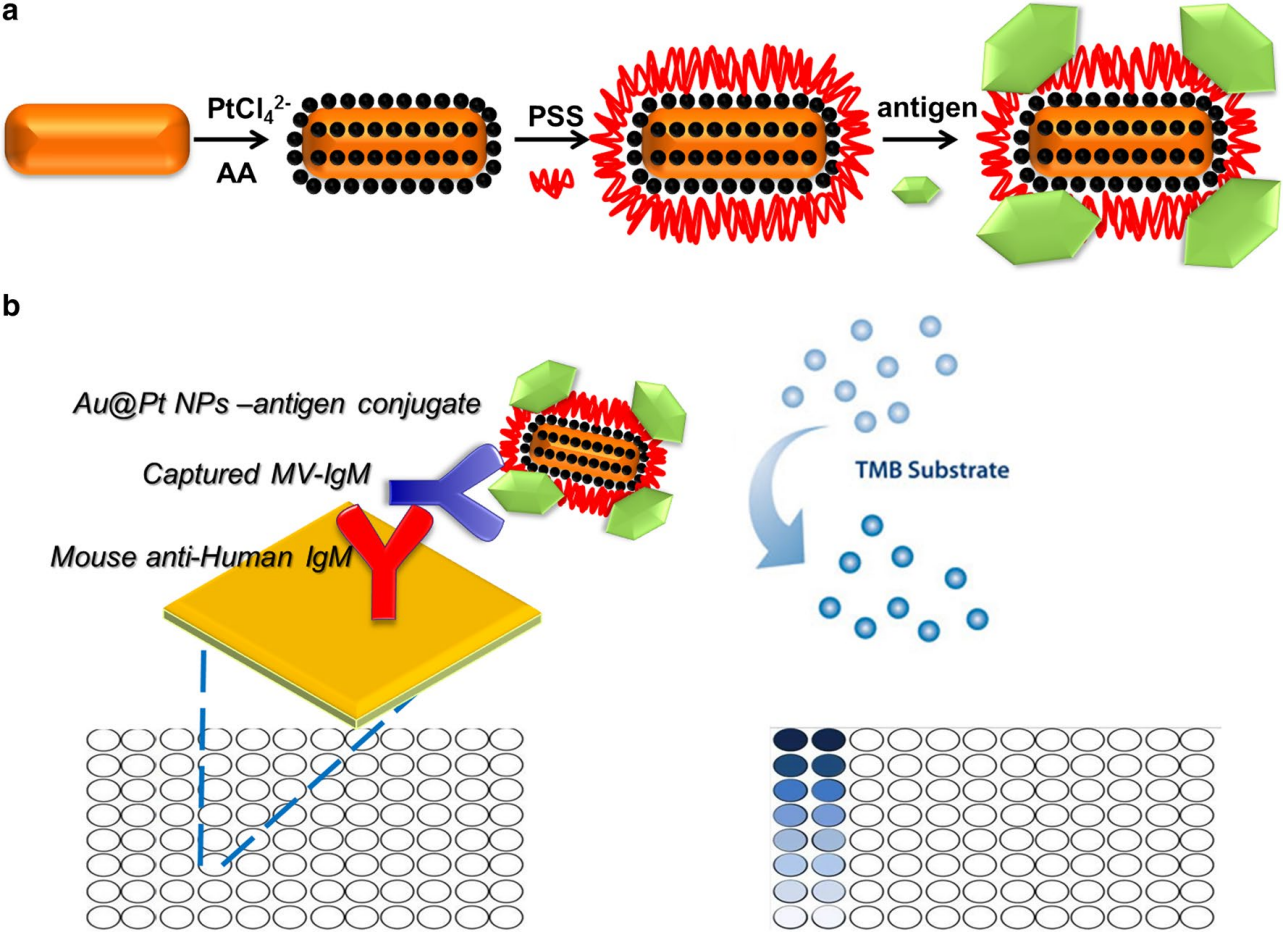
**a** Schematic representation of the synthetic procedure for Au@Pt NR-antigen conjugates. **b** The illustrated process of the immunoassay of Au@Pt NR-antigen conjugates based ELISA system

In this work, we designed a novel nanozyme-antigen conjugate and replaced HRP-antigen conjugate with it in ELISA for virus serodiagnosis. AuNR core/Pt shell nanorods (Au@Pt NRs) have an intrinsic peroxidase-like activity and are used to replace HRP. We chosen Au@Pt NRs based on the following reasons: (1) Small Pt NPs often show high catalytic activity. However, they are also easy to aggregate and thus induce the reduction of catalytic activity. A proper support is often needed to keep them in a well-dispersed state. Owing to large cohesive energy, Pt exhibits Stranski–Krastanov growth mode on gold surface. Thus, well-dispersed Pt dots can be obtained using Au as a support. Additionally, the ligand effect can further enhance the catalytic activity. (2) AuNRs are chosen as the support for the Pt nanodots due to their well-developed synthesis method and easily tailorable surface plasmonic resonance (SPR) features in the visible and near-infrared regions. The latter makes visible light-enhanced plasmonic photocatalysis possible. Taken together, The easy preparation, low cost, and robustness make Au@Pt NRs an ideal material for bioassays. Herein, in this article, we prepared nanozyme–antigen conjugates using Au@Pt NRs and measles antigen (Scheme 1a). The obtained nanozyme probe was used to monitor the specific interaction between the measles antigen and measles-specific antibody (IgM isotype), mimicking the capture-ELISA method (Scheme 1b). Then, the catalytic performance of Au@Pt NR-antigen conjugates was studied using classical enzyme kinetics. Compared with HRP-antigen conjugates, the obtained Au@Pt NR-antigen conjugates exhibited not only high peroxidase-like activity but also robustness in harsh environments, indicating that these novel nanozyme-antigen conjugates are a suitable diagnostic tool for future clinical applications under various conditions.

## Results and discussion

### Characterization of Au@Pt NRs and Au@Pt NR-antigen conjugates

**Fig. 1:**
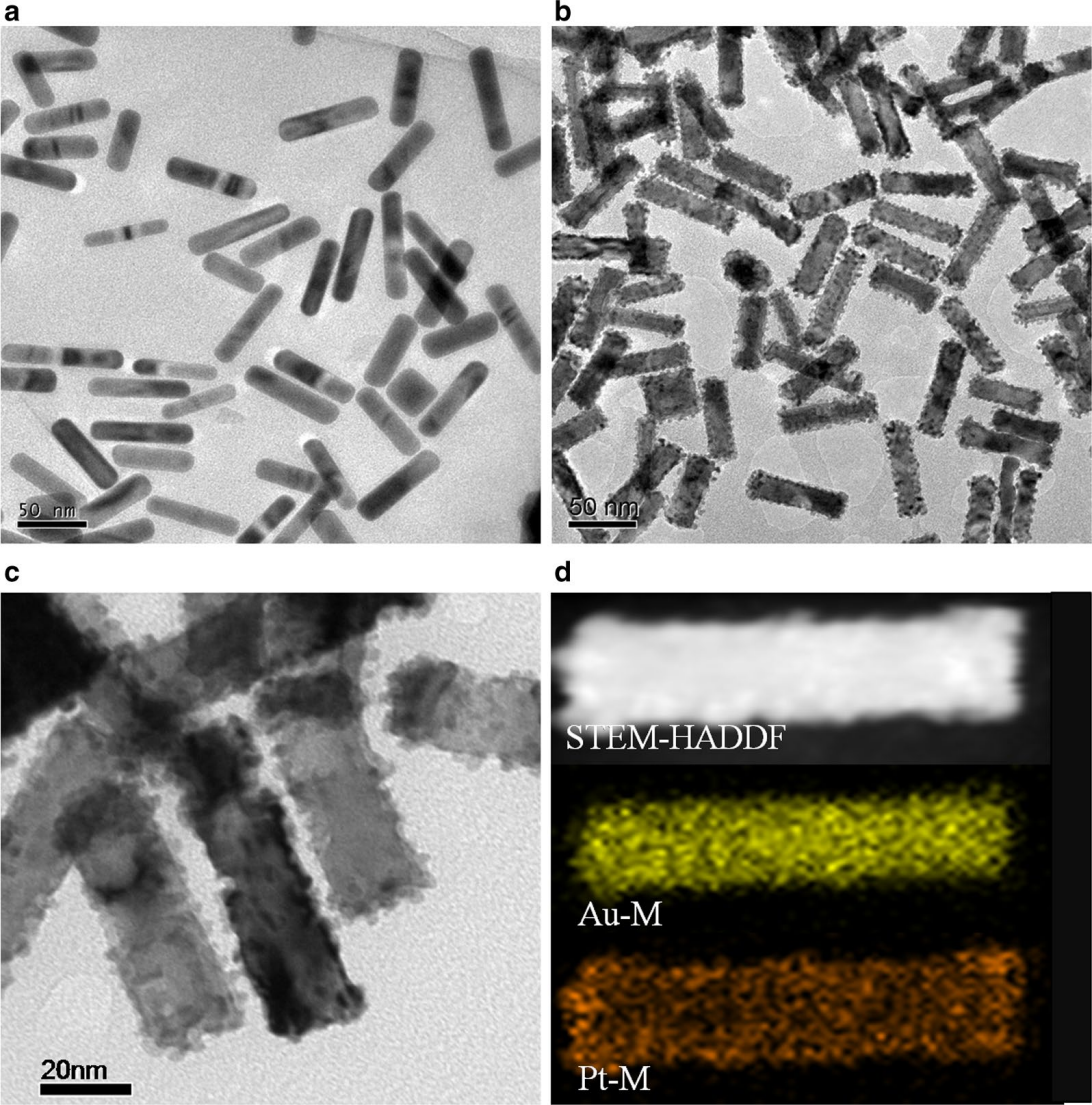
Typical TEM images of **a** Au NRs, **b** and **c** Au@Pt NRs. **d** STEM and EDX mappings of Au@Pt NRs

**Fig. 2:**
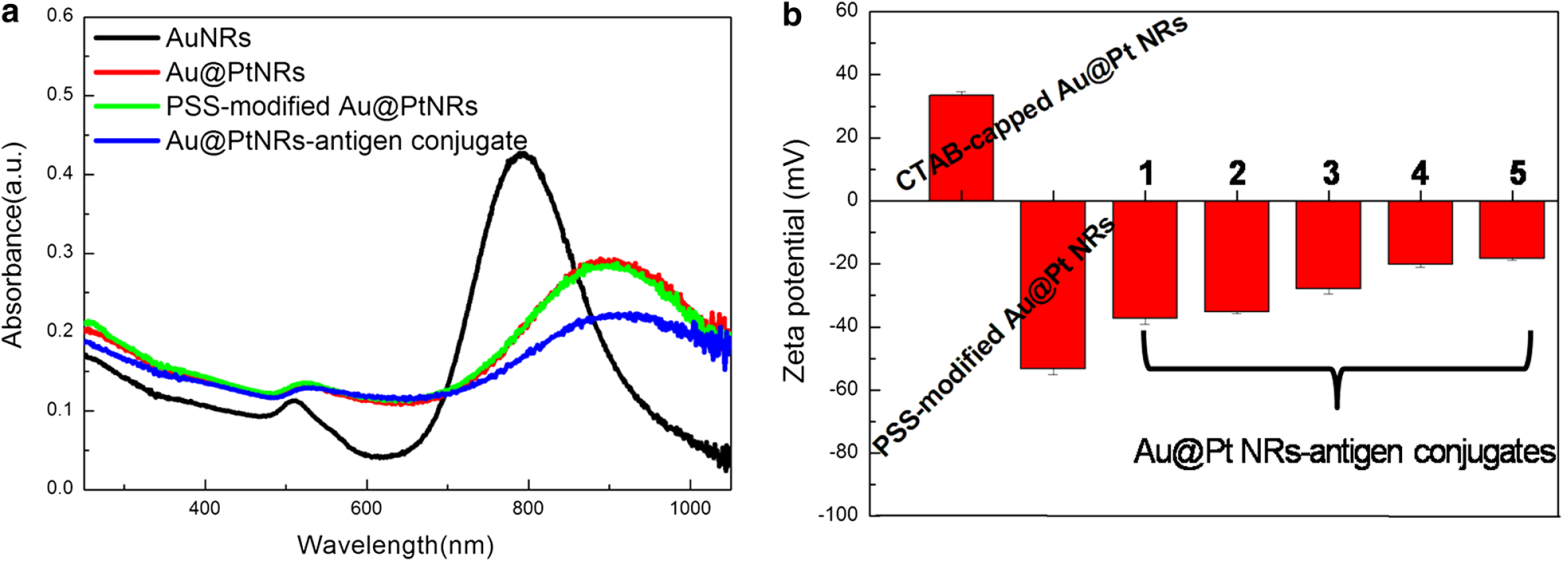
**a** UV–Vis-NIR spectra and **b** zeta potential of Au NRs, CTAB-coated Au@Pt NRs, PSS-modified Au@Pt NRs and Au@Pt NR-antigen conjugates. The numbers 1, 2, 3, 4 and 5 in **b** represent the amount of conjugating antigen. (1) 0.001 mg/mL, (2) 0.01 mg/mL, (3) 0.1 mg/mL, (4) 1 mg/mL and (5) 10 mg/mL. All error bars were calculated based on the standard deviation of three measurements

Au NRs were employed as templates to guide the growth of Pt. The average aspect ratio (AR) of the Au NRs was 3.8 (Fig. 1a). The Pt shows an island growth mode on the Au rod. Pt nanodots with sizes of 3–4 nm cover the Au rod homogeneously and form a core–shell structure, as seen from the TEM image (Fig. 1b, c) and STEM-EDX element mappings (Fig. 1d). As shown in Fig. 2a, the Au NRs exhibit a strong longitudinal SPR band with a peak at 790 nm and a weak transverse band with a peak at ~ 510 nm, respectively. Upon depositing Pt at a Pt/Au ratio of 0.25, these two bands redshift to 910 and 520 nm, respectively. The longitudinal SPR band shows a quite large redshift (100 nm) with slight damping in the intensity and an evident broadening in the width. The as-prepared Au@Pt NRs are positively charged (ζ = + 30 mV) owing to the existence of the cationic surfactant CTAB bilayer (Fig. 2b). However, CTAB-capped Au@Pt NRs are unstable and tend to form aggregates in PBS buffers or after the addition of chromogenic substrates [14]. Poly-(sodium 4-styrenesulfonate) (PSS) was coated on CTAB-capped Au@Pt NRs via electrostatic assembly and endows the NRs enhanced stability in a wide pH range. In addition, the small molecule could be directly conjugated on the surface of the nanozyme through electrostatic force. PSS modification does not lead to obvious change to the LSPR features of Au@Pt NRs (Fig. 2a). The as-prepared PSS-modified Au@Pt NRs are able to catalyze color reactions in the immunoassay while the gold NRs do not shows any peroxidase-like activity (Additional file 1: Fig. S1).

For the surface-modification method, antigen is generally conjugated on the surface of the nanozyme to provide specificity toward antibody molecules. In our work, conjugating a measles antigen to Au@Pt NRs, rather than a traditional enzyme such as HRP, enables the use of a nanozyme probe for measles virus (MV) serodiagnosis (Scheme 1). MV causes an acute, vaccine-preventable disease capable of causing epidemics. MV can be transmitted through large droplets from coughing to sneezing or direct contact with the nasal or throat secretions from an infected person [15]. Secondary infections by MV do occur and this makes the detection and monitoring of this virus very important. Serological testing methods for measles diagnosis are primarily based on the detection of specific measles immunoglobulin M (IgM) antibodies in serum samples and/or on the detection of measles RNA by real-time polymerase chain reaction (RT-PCR) in oral fluid or urine (World Health Organization, 2007). Reports have demonstrated the efficiency of detecting measles-specific IgM or IgG antibodies in human serum or plasma using ELISA techniques in indirect or capture format [16]. IgM antibodies are the first antibodies produced in the early stages of MV infection and disappear after almost 5 weeks. Therefore, they have been accepted as markers for recent or acute MV infections [17].

Sandwich-like construction of the nanozyme probe based a two-step enzyme IgM antibody capture immunoassay is illustrated in Scheme 1b. The 96-well plates were pre-coated with primary IgM antibody (mouse anti-human). During the first incubation, measles IgM antibodies present in the samples or controls bind to the solid phase. After washing, non-specific binding was removed. Subsequently, the Au@Pt NR-antigen conjugates were added into the plates to ensure binding of the nanozyme probe with the captured measles IgM antibodies. After the removal of free conjugates, the substrates TMB and H_2_O_2_ were added to initiate the color reaction, which was recorded by an ELISA reader at 450 nm.

The unspecific adsorption of antigen on Au@Pt NRs was used to form Au@Pt NRs-measles antigen conjugates. Figure 2a illustrates that antigen adsorption leads to obvious changes in the longitudinal SPR (LSPR) band of Au@Pt NRs, originating from the its high near-distance dielectric sensitivity. Zeta potential is used to predict the surface charge and stability of the NRs solution. As shown in Fig. 2b, PSS-modified NRs are negatively charged. After measles antigen adsorption, the surface charges of NRs become less negative, suggesting successful binding of antigens to the NR surface. Increasing antigen concentration leads to more antigen adsorption and thus larger increase in the Zeta potential.

### Effect of the amount of antigens and conjugation time

**Fig. 3:**
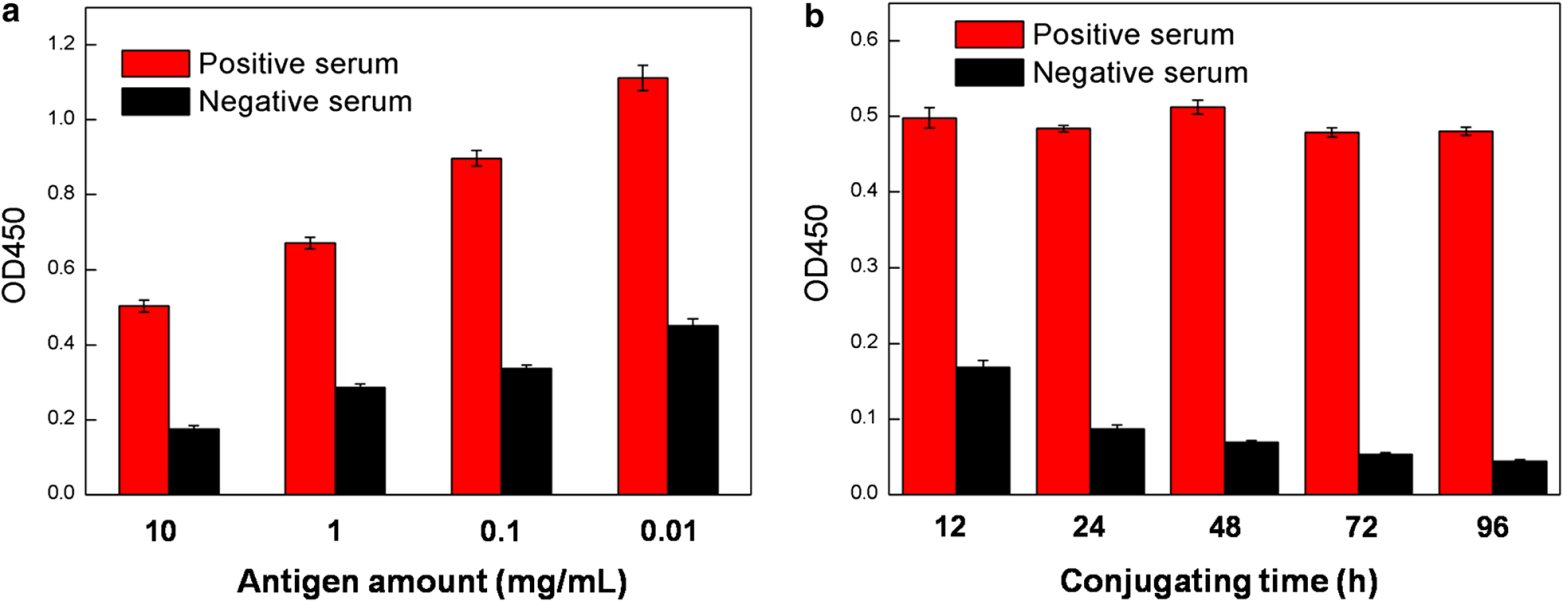
Effects of **a** amount of antigens and **b** conjugation time on the performance of Au@Pt NR-antigen conjugates in immunoassay

We then explored the performance of these conjugates in immunoassays. To study the effect of the amount of antigens used for conjugation, Au@Pt NRs were used to conjugate with varied amounts of measles antigen. The resulting conjugates were evaluated by detecting 10 mg/mL measles IgM antibodies standards. For most nanozymes, the binding sites and catalytic sites are not spatially separated; thus, modification and bioconjugation may affect the catalytic activities. In our case, the results show that increasing the number of antigen molecules conjugated onto the nanoparticles would lead to a decrease in catalytic activity, but nanoparticles harboring more antigen molecules also give a smaller value of the negative control, which is necessary to avoid a false-positive diagnosis (Fig. 3a). The high value of the negative control is largely caused by the non-specific interaction between the conjugates and the plate surface. Our results indicate that, in order to obtain a high sensitivity in nanozyme-based ELISA, the antigen amount used for the conjugation should be optimized to avoid non-specific interaction with the plate surface. Fortunately, saturated antigen conjugation could be easily achieved by using excess antigen for immobilization, and simple centrifugation could eliminate all unconjugated antigens. Furthermore, extension of the conjugation time could also reduce the value of the negative control and improve the performance of the conjugates in specific recognition (Fig. 3b).

### Kinetic analysis

**Fig. 4:**
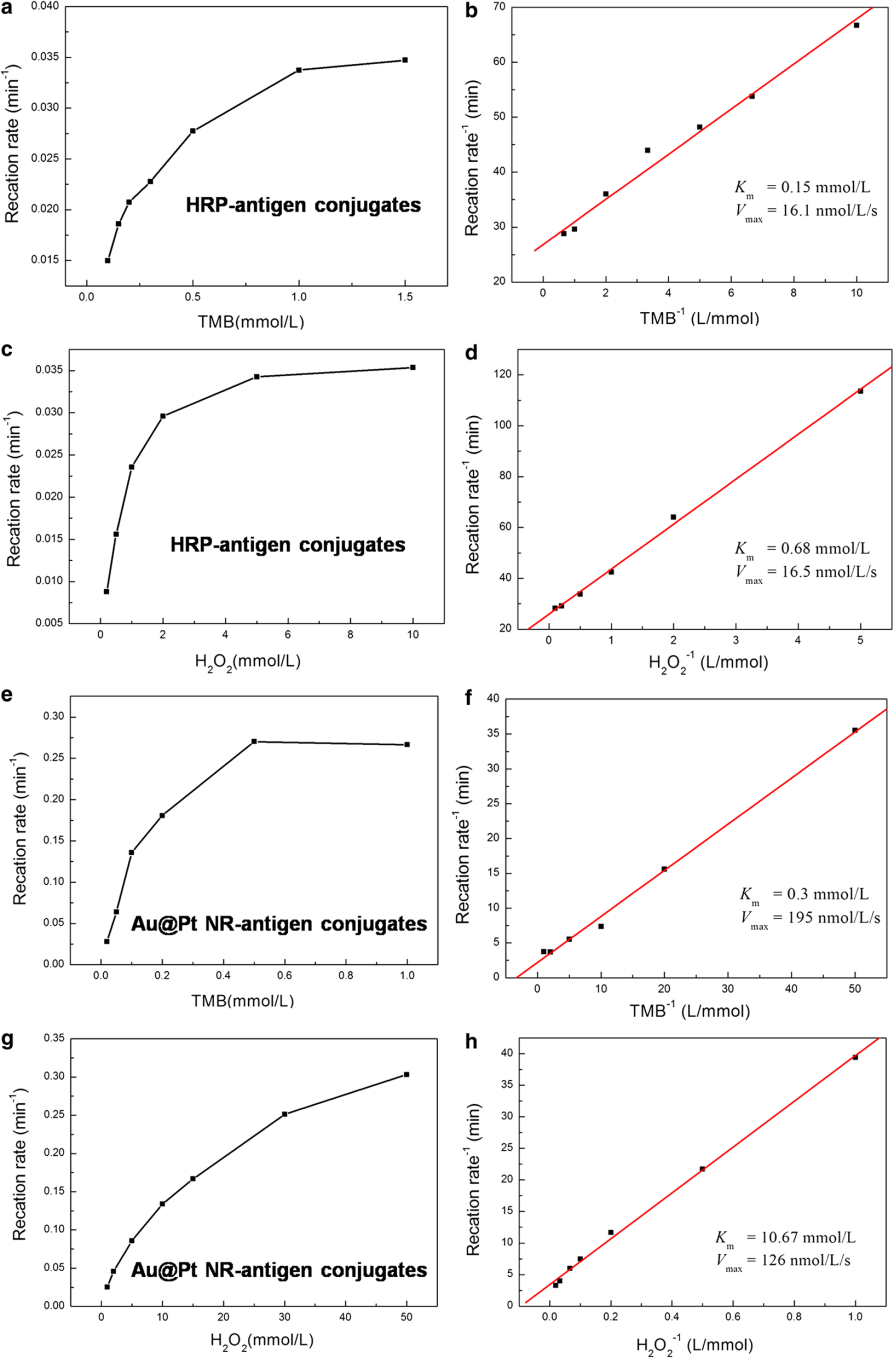
Steady state kinetic assays of HRP-antigen conjugates (**a**–**d**) and Au@Pt NR-antigen conjugates (**e**–**h**). The catalytic oxidation of TMB in the presence of H_2_O_2_ was tested. For TMB as the substrate, the H_2_O_2_ concentration was fixed at 20 mM. For H_2_O_2_ as the substrate, the TMB concentration was fixed at 1 mM

In order to evaluate the catalytic performance of Au@Pt NR-antigen conjugates, we determined the apparent enzyme kinetic parameters using TMB as the chromogenic substrate. Within a certain range of substrate concentrations, typical Michaelis–Menten curves were obtained (Fig. 4). Lineweaver–Burk plots were drawn to obtain the parameters of the Au@Pt NR-antigen conjugates and horseradish peroxidase-antigen conjugates (Table 1).

**Table 1:** Apparent kinetic parameters (*K*_m_, *V*_max_) of HRP-antigen conjugates and Au@Pt NR-antigen conjugates

Catalyst	Substrate	*K*_m_ (mM)	*V*_max_ (nM s^−1^)
HRP-antigen conjugates	TMB	0.15	16.12
HRP-antigen conjugates	H_2_O_2_	0.68	16.54
Au@Pt NR-antigen conjugates	TMB	0.30	195.17
Au@Pt NR-antigen conjugates	H_2_O_2_	10.67	125.65

Condition: at 37 °C in 0.1 M PBS buffers (pH = 5). For TMB as the substrate, the H_2_O_2_ concentration was fixed at 20 mM. For H_2_O_2_ as the substrate, the TMB concentration was fixed at 1 mM

For peroxidase-like activity, *K*_m_ values of the Au@Pt NR-antigen conjugates for TMB are in the micromolar range, similar to the values of HRP-antigen conjugates. For natural enzymes, *K*_m_ is an indicator of enzyme affinity to the substrate. A larger *K*_m_ represents a lower affinity, whereas a smaller value suggests a higher affinity. Compared with a previous report [14], Au@Pt NR-antigen conjugates show a higher *K*_m_ than Au@Pt NRs, whose *K*_m_ was 0.026 μM. Two possible sources may lead to the reduced affinity. First, adsorption of antigens occupy some binding sites for TMB. Besides, the formed antigen layer itself has a low affinity to TMB. Despite the reduced substrate affinity, the Au@Pt NR-antigen conjugates show a high peroxidase-like activity due to more catalytic sites provided by the larger surface area of nanoparticles.

### Catalytic stability of Au@Pt NR-antigen conjugates and HRP-antigen conjugates against temperature and pH

**Fig. 5:**
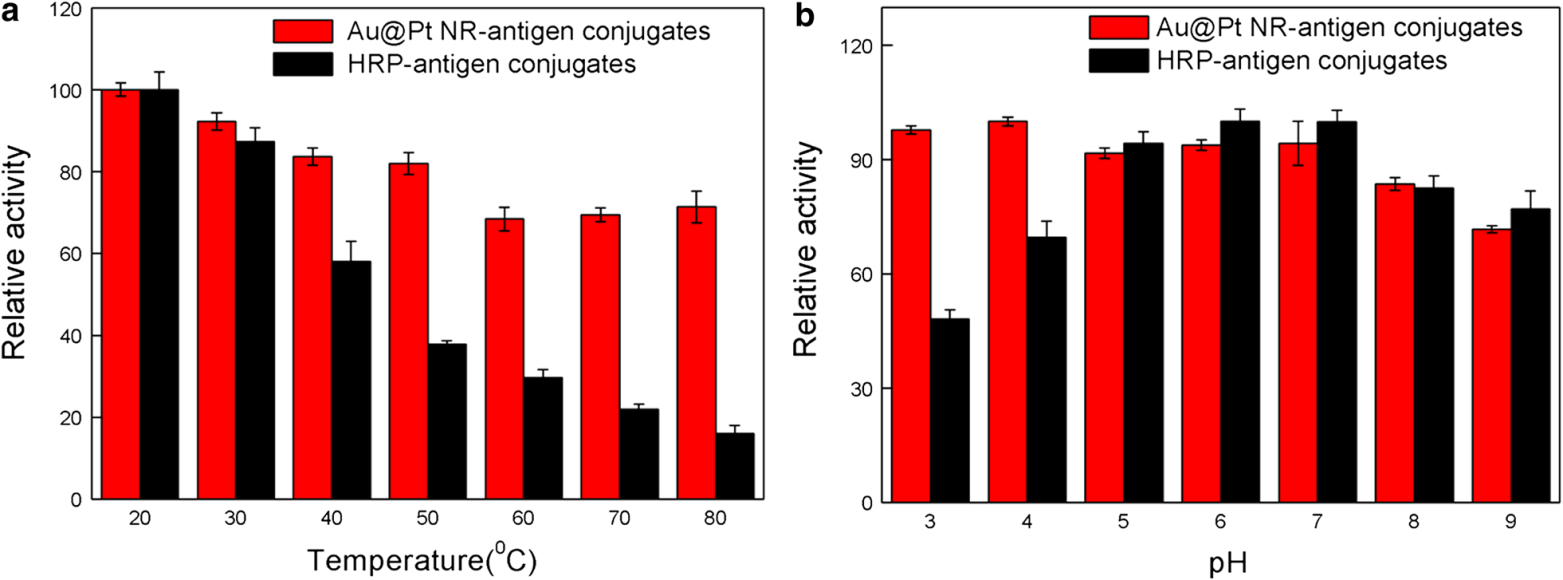
Comparison of the stability of HRP-antigen conjugates and Au@Pt NR-antigen conjugates. **a** HRP-antigen conjugates and Au@Pt NR-antigen conjugates were treated at a wide range of temperatures between 20 and 80 °C for 3 h, and the peroxidase activity was measured under standard conditions. **b** HRP-antigen conjugates and Au@Pt NR-antigen conjugates were treated in media with a range of pH from 3 to 9 for 3 h, and then their peroxidase activities were measured under standard conditions

To evaluate the robustness of the peroxidase-like activities, both HRP-antigen conjugates and Au@Pt NR-antigen conjugates were first incubated for 3 h in a range of temperatures (20–80 °C) or treated in aqueous media with a range of pH values of 3–9. Then, their peroxidase-like performance was examined under the standard conditions of pH 5.0 and 37 °C. As presented in Fig. 5a, the robustness of Au@Pt NR-antigen conjugates towards a wide range of temperature is ambiguously illustrated. In contrast, the peroxidase-like activity of HRP-antigen conjugates quickly decreases at increased treatment temperatures. The peroxidase-like activity of Au@Pt NR-antigen conjugates and HRP after treatment over a wide pH range from 3 to 9 is presented in Fig. 5b. Unlike HRP-antigen conjugates, which lose activity after treatment at pH lower than 5, the peroxidase-like activity of treated Au@Pt NR-antigen conjugates is more or less unchanged at all employed pH values. Thus, the high chemical stability of Au@Pt NR-antigen conjugates under harsh reaction conditions is demonstrated.

### Optimal substrates concentration, conjugate concentration, reaction time, temperature, and pH

To achieve optimal analytical performance of the Au@Pt NR-antigen conjugates, the effects of substrate concentration, conjugate concentration, reaction time, temperature, and pH were first studied in a TMB-H_2_O_2_ colorimetric system (Additional file 1: Fig. S2). For the effect of TMB or H_2_O_2_ concentration on the absorbance, they show a similar trend with the increase in substrate TMB concentration from 0.1 to 0.5 mM, and H_2_O_2_ concentration from 5 to  30 mM (Additional file 1: Fig. S2A, B). When increasing the Au@Pt NR-antigen conjugate concentration from 0.025 to 0.125 nM, a linear relationship is also observed (Additional file 1: Fig. S2C). The absorbance at 450 nm shows a linear increase with time within 10 min (Additional file 1: Fig. S2D). The peroxidase-like activity of Au@Pt NR-antigen conjugates was also evaluated at various temperatures of 20–60 °C, with the highest catalytic activity at approximately 30 °C, and decayed performance is observed at either elevated temperatures or decreased temperatures (Additional file 1: Fig. S2E). By varying the pH from 3  to  9, the maximized catalytic activity of Au@Pt NR-antigen conjugates is found at pH values between 4 and 5 (Additional file 1: Fig. S2F), which is very close to that of HRP. According to the above results, 0.125 nM Au@Pt NR-antigen conjugates, 0.5 mM TMB, 20 mM H_2_O_2_, 37 °C and pH 5 were separately selected in the ELISA.

### Application of biomedical assay

**Fig. 6:**
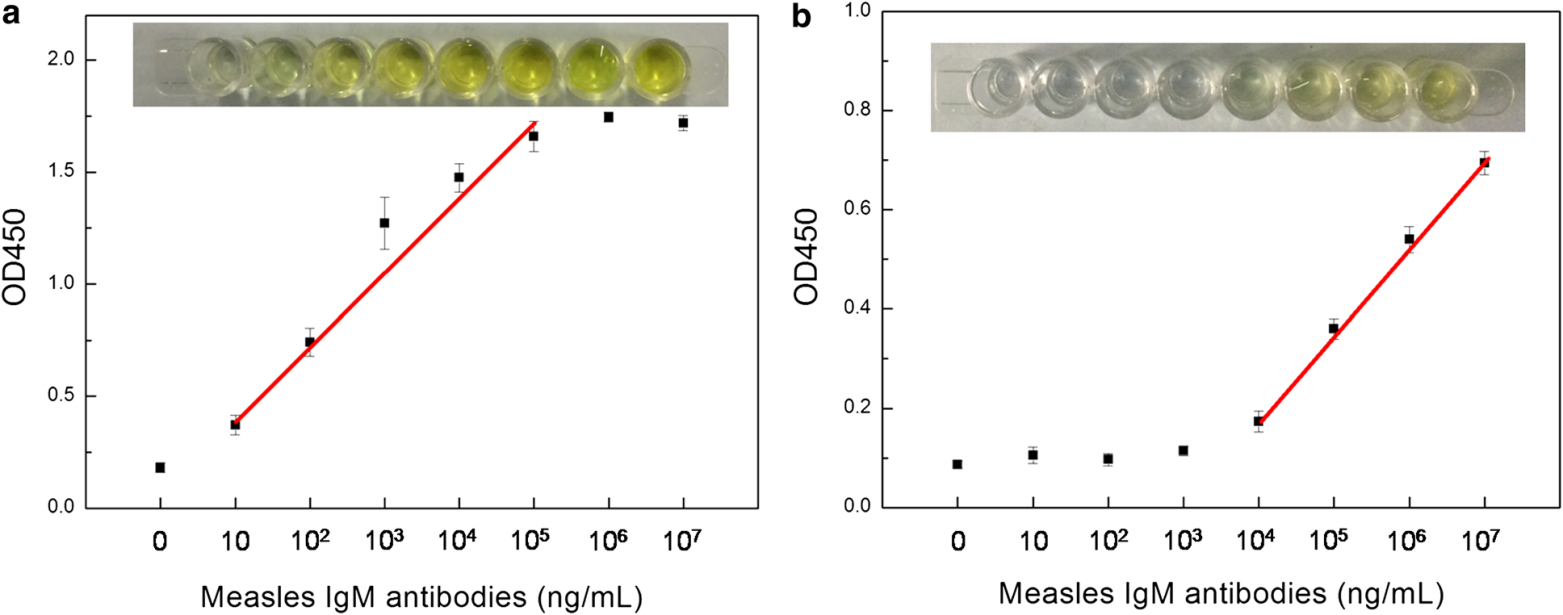
Immunosorbent assay of measles IgM antibodies: **a** Au@Pt NR-antigen conjugate-based ELISA; **b** HRP-antigen conjugate-based ELISA. The relation of the mean absorbance intensity at 450 nm and measles IgM antibodies concentration. All error bars were calculated based on the standard deviation of three measurements. The insets are the corresponding color in the well

As shown in Fig. 6, the concentration of measles IgM antibodies in the simulated sample displayed a linear relationship in the oxidation reaction of TMB for both HRP-antigen and Au@Pt NR-antigen conjugate-based immunoassay. The linear range of the Au@Pt NR-antigen conjugate-based ELISA was 10–10^4^ ng/mL. Impressively, the detection limit of Au@Pt NR-antigen conjugate-based immunoassay was 10 ng/mL, which is three orders of magnitude higher than that of the HRP-antigen system. The results demonstrated that the system would have excellent capability in response to changes of the actual serum samples. A total of 90 serum samples (60 MV-negative and 30 MV-positive, which have been diagnosed by commercial ELISA) were measured by Au@Pt NR-antigen conjugate-based ELISA. When the cutoff value was defined as three times the blank signal, the Au@Pt NR-antigen conjugate-based ELISA gave completely consistent results with commercial ELISA for all of the samples.

**Fig. 7:**
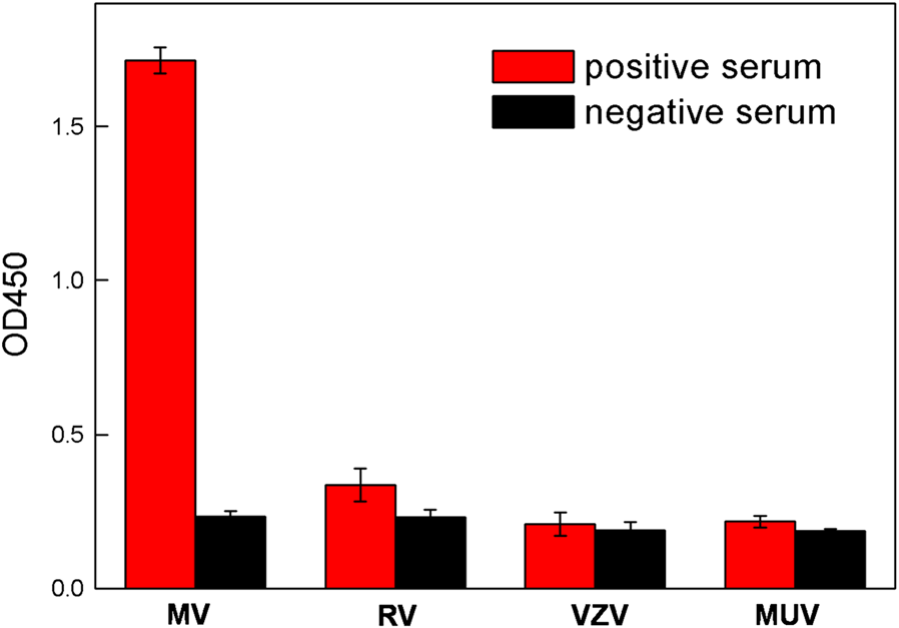
Specificity of MV, rubella virus (RV), varicella-zoster virus (VZV) and mumps virus (MUV) positive and negative serum using Au@Pt NR-antigen conjugate-based ELISA

Furthermore, the specificity of the ELISA system for MV positive serum was also investigated by comparing with other infectious viruses. As shown in Fig. 7, stronger optical densities were acquired for MV-positive serum. And only very weak signals appeared for the other positive or negative serum. The results demonstrated that the measles IgM antibodies can be effectively recognized by the proposed ELISA system with high specificity.

## Conclusions

In summary, herein, we studied the peroxidase-like activity of Au@Pt NR-antigen conjugates and its application in the detection of measles IgM antibodies through colorimetric immunoassay. The kinetic results showed that Au@Pt NR-antigen conjugates exhibited *K*_m_ value to TMB similar to that of HRP-antigen conjugates. Moreover, the large surface area provided the most catalytic sites, resulting in stronger peroxidase-like activity compared to that of natural HRP enzyme. An almost constant peroxidase-like activity was also found for fresh Au@Pt NR-antigen conjugates and treated Au@Pt NR-antigen conjugates at different temperatures (20–80 °C) or pH values (3–9). Hence, the accuracy and reliability of Au@Pt NR-antigen conjugate-based immunoassay were expected, as illustrated for the specific detection of measles IgM antibodies. The high sensitivity of Au@Pt NR-antigen conjugate-based immunoassay with a detection limit of 10 ng/mL was three orders of magnitude higher than that of the HRP-antigen conjugates system. The highly sensitive peroxidase-like activity of Au@Pt NR-antigen conjugates, along with their catalytic stability and robustness, can facilitate their utilization in biochemical assay and clinical diagnosis. Moreover, it can be conceived that a similar strategy could be applicable to construct other nanozyme-molecule conjugates for use in immunoassays.

## Methods

### Materials

Sodium borohydride (NaBH_4_), cetylmethylammonium bromide(CTAB), poly(styrene sulfonic acid) sodium salt (PSS), chloroauric acid (HAuCl_4_·3H_2_O), potassium tetrachloroplatinate(II) (K_2_PtCl_4_), copper chloride (CuCl_2_), silver nitrate (AgNO_3_), l-ascorbic acid (AA), 30% H_2_O_2_, and 3,3′,5,5′-tetramethylbenzidinedihydrochloride (TMB) were all purchased from Alfa Aesar (USA) and used as received. Measles-antigen was purchased from Beier Bioengineering Company (China). Rabbit anti-human IgM antibodies coated plate, HRP-antigen conjugates, and positive and negative serum samples (ELISA kit) were purchased from Haitai Biopharmaceutical Company (China). Milli-Q water (18 MΩ cm) was used for all solution preparations.

### Synthesis of gold nanorods (NRs)

Au NRs were synthesized using a seed-mediated growth procedure. CTAB-capped Au seeds were synthesized by chemical reduction of HAuCl_4_ with NaBH_4_. CTAB (7.5 mL, 0.1 M) was mixed with HAuCl_4_ (100 μL, 24 mM), diluted with water to 9.4 mL, and stirred with a magnetic stirrer. Then, ice-cold NaBH_4_ (0.6 mL, 0.01 M) was added. The solution color immediately turned from bright yellow to brown, indicating the formation of seeds. The Au seeds were used within 2–5 h. A 120 μL aliquot of the seed solution was added to the growth solution consisting of CTAB (100 mL, 0.1 M), HAuCl_4_ (2.04 mL, 24 mM), AgNO_3_ (1.05 mL, 10 mM), H_2_SO_4_ (2 mL, 0.5 M) and AA (800 μL, 0.1 M) to initiate the growth of Au NRs. After 12 h, the reaction was stopped. The obtained Au NRs were purified by centrifuging the solution at 12,000 rpm for 5 min twice. The precipitate was collected and redispersed in deionized water.

### Synthesis of Au@Pt NRs

Three samples of the purified Au NR solutions (1 mL) were mixed with 62.5 μL of 2 mM PtCl_4_^2−^ aqueous solution. Then, 12.5 μL of 0.1 M AA was added, and the total solution volume was diluted to 2 mL. The mixture was shaken vigorously and then placed in a 30 °C water bath for 30 min. Within several minutes, the color of the solution changed from pink to red to dark gray, suggesting the formation of a Pt shell. Then, 1 mL of 0.1 M CTAB was added.

### Modification of the Au@Pt NRs with PSS

CTAB-coated nanorod solution (1 mL, Au@Pt NRs) was centrifuged at 12,000 rpm for 10 min, and the precipitate was dispersed in 0.5 mL of PSS aqueous solution (2 mg/mL containing 6 mM NaCl). Then, the solution was stirred magnetically for 3 h. After that, it was centrifuged at 12,000 rpm for 10 min, and the precipitate was redispersed in water.

### Preparation of Au@Pt NR-antigen conjugates

PSS-coated Au@Pt NRs solution (50 µL, 5 nM) was first dispersed into 1 mL of PBS buffer (0.1 M, pH 7.4). Then, 50 μL of 10 mg/mL measles antigen was added to the above Au@Pt NRs solution and incubated at 37 °C for 96 h. After incubation, the mixture was centrifuged at 12,000 r/min for 5 min twice. Then, the precipitate was collected and redispersed in 100 μL of PBS buffer (0.1 M, pH 7.4). Finally, it was dispersed in PBS buffer (pH 7.4) at a concentration of 2.5 nM.

### Kinetic analysis

The apparent kinetic parameters were obtained by using the Lineweaver–Burk double reciprocal plot:

$$ \frac{1}{\text{v}} = \left( {\frac{{K_{m} }}{{V_{\text{max} } }}} \right)\frac{1}{[c]} + \frac{1}{{V_{\text{max} } }} $$

where v is the initial velocity, *V*_max_ is the maximal reaction velocity, and [c] is the concentration of substrate.

The reaction kinetics for the catalytic oxidation of TMB were studied by recording the absorption spectra at 0.25-min intervals using a Varian Cary 50 in kinetics mode. Steady-state kinetic assays were carried out at 30 °C in 0.1 M PBS buffer (pH 5) in the presence of NRs (0.0125 nM). To investigate the peroxidase-like activity of the HRP-antigen conjugates and Au@Pt NR-antigen conjugates, the catalytic oxidation of TMB in the presence of H_2_O_2_ was tested. For TMB as the substrate, the H_2_O_2_ concentration was fixed at 20 mM. For H_2_O_2_ as the substrate, the TMB concentration was fixed at 1 mM.

### Detection of measles IgM antibodies by ELISA

ELISA detection of measles IgM antibodies was performed in 96-well polystyrene plates. Each well of the 96-well plates was pre-coated with mouse anti-human IgM antibodies. First, each well was blocked with 5% BSA (diluted in PBS, pH 7.4) for 1 h at 37 °C to avoid non-specific interaction with the plate surface. Then, the plates were washed three times with PBST buffer (pH 7.4). After that, 100 μL of negative control, positive control or diluted sample was added to the plate and incubated at 37 °C for 1 h. The plates were washed three times with PBST buffer (pH 7.4) to remove the unbound measles IgM antibodies. Then, 100 μL of Au@Pt NR-antigen conjugates was added to each well and incubated for 0.5 h at 37 °C. The plates were washed five times with PBST buffer (pH 7.4) to remove the unbound Au@Pt NR-antigen conjugates. The color development was initiated by adding 100 μL of substrate solution (0.5 mM TMB, 20 mM H_2_O_2_ in PBS buffer, pH 5) into each well. The reaction was stopped after 10 min using 50 μL of 2 M H_2_SO_4_. Absorbance was measured at 450 nm. The clinical serum sample was selected from patients with clinical signs of measles, or patients who had been exposed to measles. The clinical serum experiment was checked with the positive control, negative control and the blank. Buffer solution was used as the blank.

### Characterizations

UV–Vis-NIR extinction spectra were obtained from a Varian Cary 50. Transmission electron microscopy (TEM) was performed on a Tecnai G2 T20 S-TWIN (T20). Scanning transmission electron microscopy (STEM) and energy dispersive X-ray analysis (EDX) element mappings were conducted with a Tecnai G2 F20 U-Twin microscope using copper grids. The zeta potential data were obtained from a Delsa Nano C (Beckman Coulter). ELISA data was obtained on an Infinite™ M200.

## Additional file

**Additional file 1: Fig. S1.** Typical photographs of TMB–H_2_O_2_ solution (left) TMB–H_2_O_2_-Au NRs (middle) and TMB–H_2_O_2_-Au@Pt NRs (right). Reaction conditions: 0.5 mM TMB, 20 mM H_2_O_2_ and 0.125 nM Au NRs/Au@Pt NRs. **Fig. S2.** Effects of substrates concentration (TMB), substrates concentration (H_2_O_2_), conjugate concentration (Au@Pt NR-antigen conjugates), temperature, reaction time and pH on catalytic activity of the Au@Pt NR-antigen conjugates. Reaction conditions: (A) 0.125 nM Au@Pt NRs, 20 mM H_2_O_2_, (B) 0.125 nM Au@Pt NRs and 0.5 mM TMB, (C) 0.5 mM TMB and 20 mM H_2_O_2_, (D-F) 0.125 nM Au@Pt NRs, 0.5 mM TMB and 20 mM H_2_O_2_.
