# Correction to: Highly sensitive and robust peroxidase-like activity of Au–Pt core/shell nanorod-antigen conjugates for measles virus diagnosis

**DOI:** 10.1186/s12951-018-0435-1

**Published:** 2019-01-10

**Authors:** Lin Long, Jianbo Liu, Kaishun Lu, Tao Zhang, Yunqing Xie, Yinglu Ji, Xiaochun Wu

**Affiliations:** 1 College of Opto-electronic Engineering, Zaozhuang University, Zaozhuang, 277160 China; 2 Zaozhuang Municipal Center for Disease Control and Prevention, Zaozhuang, 277100 China; 3 CAS Key Laboratory of Standardization and Measurement for Nanotechnology, National Center for Nanoscience and Technology, Beijing, 100190 China

## Correction to: J Nanobiotechnol (2018) 16:46 10.1186/s12951-018-0371-0

After publication of the original article [1], an error was noted in the author affiliation. Lin Long is also affiliated to the College of Opto-electronic Engineering, Zaozhuang University, Zaozhuang, China, which is her first affiliation.
