# Supplementary material for: Highly sensitive and robust peroxidase-like activity of Au–Pt core/shell nanorod-antigen conjugates for measles virus diagnosis
Source: J Nanobiotechnology. 2018 May 2;16:46. doi: 10.1186/s12951-018-0371-0 (PMC5930499; doi:10.1186/s12951-018-0371-0)
Supplement: Supplementary file 1 — Additional file 1: Fig. S1. Typical photographs of TMB–H2O2 solution (left) TMB–H2O2-Au NRs (middle) and TMB–H2O2-Au@Pt NRs (right). Reaction conditions: 0.5 mM TMB, 20 mM H2O2 and 0.125 nM Au NRs/Au@Pt NRs. Fig. S2. Effects of substrates concentration (TMB), substrates concentration (H2O2), conjugate concentration (Au@Pt NR-antigen conjugates), temperature, reaction time and pH on catalytic activity of the Au@Pt NR-antigen conjugates. Reaction conditions: (A) 0.125 nM Au@Pt NRs, 20 mM H2O2, (B) 0.125 nM Au@Pt NRs and 0.5 mM TMB, (C) 0.5 mM TMB and 20 mM H2O2, (D-F) 0.125 nM Au@Pt NRs, 0.5 mM TMB and 20 mM H2O2. [file 12951_2018_371_MOESM1_ESM.docx]

**Supplementary Material**

**Highly sensitive and robust peroxidase-like activity of Au-Pt core/shell nanorod-antigen conjugates for measles viurs diagnosis**

**Lin Long ^1,†^, Jianbo Liu^2^*^,†^ , Kaishun Lu^1^, Tao Zhang^1^, Yunqing Xie^1^, Yinglu Ji^3^ and Xiaochun Wu^3^***

*^1^* *Zaozhuang Municipal Center for Disease Control and Prevention, Zaozhuang 277100, China*

*^2^* *College of Opto-electronic Engineering, Zaozhuang University, Zaozhuang 277160, China*

*^3^* *CAS Key Laboratory of Standardization and Measurement for Nanotechnology, National Center for Nanoscience and Technology, Beijing 100190, China*

*^†^These authors contributed equally to this work*

** Corresponding author (email: linyibm@163.com (J. Liu), wuxc@nanoctr.cn (X. Wu))*


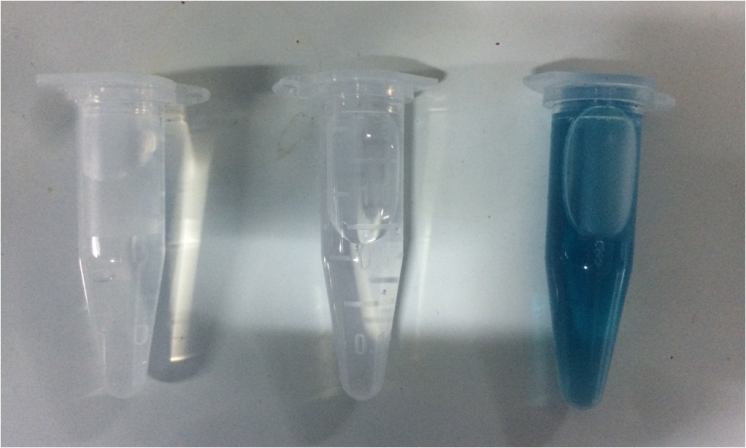


**Fig. S1.** Typical photographs of TMB–H_2_O_2_ solution (left) TMB–H_2_O_2_-Au NRs (middle) and TMB–H_2_O_2_-Au@Pt NRs (right). Reaction conditions: 0.5mM TMB, 20mM H_2_O_2_ and 0.125 nM Au NRs/Au@Pt NRs.


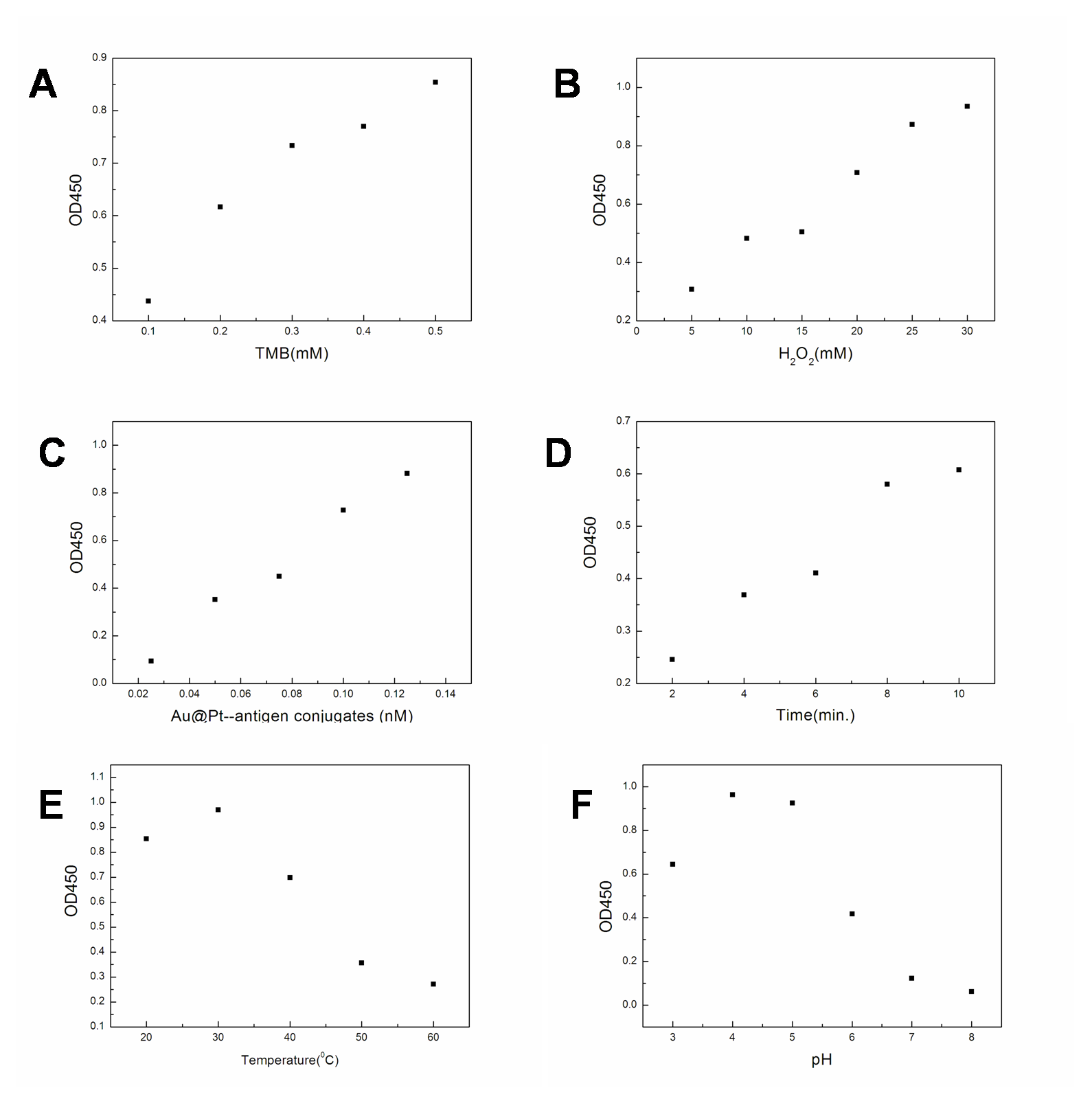


**Fig. S2.** Effects of substrates concentration (TMB), substrates concentration (H_2_O_2_), conjugate concentration (Au@Pt NR-antigen conjugates), temperature, reaction time and pH on catalytic activity of the Au@Pt NR-antigen conjugates. Reaction conditions: (A) 0.125 nM Au@Pt NRs, 20mM H_2_O_2_, (B) 0.125 nM Au@Pt NRs and 0.5 mM TMB, (C) 0.5mM TMB and 20mM H_2_O_2_, (D-F) 0.125 nM Au@Pt NRs, 0.5 mM TMB and 20mM H_2_O_2_.
